# What Is Resistance? Impact of Phenotypic versus Molecular Drug Resistance Testing on Therapy for Multi- and Extensively Drug-Resistant Tuberculosis

**DOI:** 10.1128/AAC.01550-17

**Published:** 2018-01-25

**Authors:** Jan Heyckendorf, Sönke Andres, Claudio U. Köser, Ioana D. Olaru, Thomas Schön, Erik Sturegård, Patrick Beckert, Viola Schleusener, Thomas A. Kohl, Doris Hillemann, Danesh Moradigaravand, Julian Parkhill, Sharon J. Peacock, Stefan Niemann, Christoph Lange, Matthias Merker

**Affiliations:** a Division of Clinical Infectious Diseases, Research Center Borstel, Borstel, Germany; b German Center for Infection Research (DZIF), Partner site Hamburg-Lübeck-Borstel, Borstel, Germany; c International Health/Infectious Diseases, University of Lübeck, Lübeck, Germany; d Division of Mycobacteriology (National Tuberculosis Reference Laboratory), Research Center Borstel, Borstel, Germany; e Department of Genetics, University of Cambridge, Cambridge, United Kingdom; f Department of Infectious Diseases and Clinical Microbiology, Kalmar County Hospital, Kalmar, Sweden; g Department of Clinical and Experimental Medicine, Division of Medical Microbiology, Linköping University, Linköping, Sweden; h Clinical Microbiology, Department of Translational Medicine, Lund University, Malmö, Sweden; i Division of Molecular and Experimental Mycobacteriology, Research Center Borstel, Borstel, Germany; j Wellcome Trust Sanger Institute, Hinxton, United Kingdom; k London School of Hygiene & Tropical Medicine, London, United Kingdom; l Department of Medicine, Karolinska Institute, Stockholm, Sweden; m Department of Medicine, University of Namibia School of Medicine, Windhoek, Namibia

**Keywords:** Mycobacterium tuberculosis, antibiotic resistance, molecular genetics

## Abstract

Rapid and accurate drug susceptibility testing (DST) is essential for the treatment of multi- and extensively drug-resistant tuberculosis (M/XDR-TB). We compared the utility of genotypic DST assays with phenotypic DST (pDST) using Bactec 960 MGIT or Löwenstein-Jensen to construct M/XDR-TB treatment regimens for a cohort of 25 consecutive M/XDR-TB patients and 15 possible anti-TB drugs. Genotypic DST results from Cepheid GeneXpert MTB/RIF (Xpert) and line probe assays (LPAs; Hain GenoType MTBDR*plus* 2.0 and MTBDR*sl* 2.0) and whole-genome sequencing (WGS) were translated into individual algorithm-derived treatment regimens for each patient. We further analyzed if discrepancies between the various methods were due to flaws in the genotypic or phenotypic test using MIC results. Compared with pDST, the average agreement in the number of drugs prescribed in genotypic regimens ranged from just 49% (95% confidence interval [CI], 39 to 59%) for Xpert and 63% (95% CI, 56 to 70%) for LPAs to 93% (95% CI, 88 to 98%) for WGS. Only the WGS regimens did not contain any drugs to which pDST showed resistance. Importantly, MIC testing revealed that pDST likely underestimated the true rate of resistance for key drugs (rifampin, levofloxacin, moxifloxacin, and kanamycin) because critical concentrations (CCs) were too high. WGS can be used to rule in resistance even in M/XDR strains with complex resistance patterns, but pDST for some drugs is still needed to confirm susceptibility and construct the final regimens. Some CCs for pDST need to be reexamined to avoid systematic false-susceptible results in low-level resistant isolates.

## INTRODUCTION

Tuberculosis (TB) is a leading cause of morbidity and mortality worldwide (1). Although the global incidence of TB has been slowly declining, the emergence of multidrug-resistant TB (MDR-TB), defined as resistance to rifampin and isoniazid, challenges TB control (1). Extensively drug-resistant TB (XDR-TB), defined as MDR-TB and resistance to at least one fluoroquinolone (e.g., ofloxacin, levofloxacin, or moxifloxacin; World Health Organization [WHO] group A) and any second-line injectable drug (SLID; amikacin, kanamycin, or capreomycin; WHO group B) has been reported in 117 countries (1).

Therapy of M/XDR-TB is complex and requires a long duration of treatment with a combination of at least four drugs, often leading to adverse events and poor treatment outcomes (2, 3). Moreover, the initiation of appropriate therapy is often delayed due to the low growth rate of Mycobacterium tuberculosis complex isolates, which means that phenotypic drug susceptibility testing (pDST) can take weeks to months (4, 5). To accelerate this rate-limiting step, a number of genotypic DST assays that detect resistance mutations have been endorsed by the WHO (6). The Cepheid GeneXpert (Xpert) is an automated point-of-care assay with a high diagnostic accuracy for rifampin resistance detection, providing results within 1.5 h (7). Line probe assays (LPAs; e.g., Hain GenoType MTBDR*plus* 2.0 and MTBDR*sl* 2.0) also can be performed directly from sputum to provide results within 1 to 2 days with a high diagnostic accuracy for resistance to isoniazid, rifampin, fluoroquinolones, and SLIDs (6). Because these assays only target a limited number of resistance variants, their sensitivity compared with that of pDST is limited. Whole-genome sequencing (WGS) theoretically can overcome this shortcoming by interrogating the entire genetic repertoire (4, 5, 8). Nevertheless, the utility of WGS is currently limited by the need for expensive equipment, highly trained personnel, and complex bioinformatic procedures. Moreover, WGS requires an initial culture, which introduces a delay compared with the aforementioned targeted assays (6, 9). More fundamentally, there is a lack of understanding of the genetic basis of antibiotic resistance, which complicates the interpretation of WGS data (10).

However, it is important to appreciate that discrepancies observed between pDST and genotypic methods are not exclusively due to problems related to the interpretation of the genotype (6). Instead, evidence is mounting that some critical concentrations (CCs), which are set by the Clinical and Laboratory Standards Institute (CLSI) and/or WHO and define resistance on a phenotypic level, are higher than the epidemiological cutoff values (ECOFFs), which represent the highest concentration of the wild-type MIC distribution (6, 11–15). As a result, some isolates with elevated MICs compared to the ECOFF due to known mutations are classified as susceptible even though limited pharmacokinetic/pharmacodynamics or clinical outcome data exist that these isolates are still treatable (6, 12, 13, 16).

Therefore, this study had two main goals. First, we compared the utility of genotypic methods (Xpert, LPAs, and WGS) with pDST to design M/XDR regimens using standardized algorithms. Second, we analyzed whether discrepancies between the various methods were due to flaws in pDST or the genotype.

## RESULTS

### Patient cohort.

Twenty patients with MDR-TB and 5 with XDR-TB admitted to the Medical Clinic of the Research Center Borstel (Germany) were enrolled (see Table S1 in the supplemental material).

### Comparison of M/XDR-TB regimens based on pDST with molecular methods.

**FIG 1:**
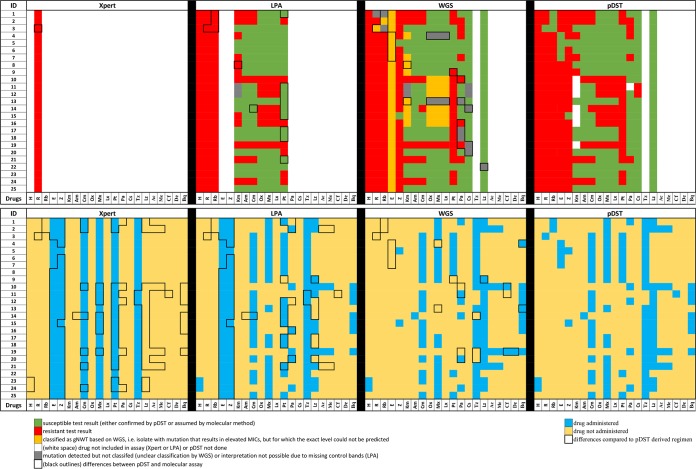
Comparison of pDST, Xpert, LPA, and WGS results and corresponding regimens. (Upper) Results for pDST and molecular methods (Xpert, LPAs, and WGS) for 25 M. tuberculosis isolates from patients with M/XDR-TB. Test results denoting either confirmed phenotypic susceptibility or assumed susceptibility based on genotypic methods are shown in green, those denoting resistance are in red, gNWT variants with elevated MICs are in orange, and mutations with unclear effects are in gray. Differences between Xpert, LPA, and WGS results compared to those of the pDST are outlined by black margins (both gNWT and unclear variants were assumed to be resistant for the purposes of designing the regimens and results between DST methods). (Lower) Standard algorithm-derived treatment regimens based on respective results of pDST, LPAs, WGS, and Xpert. Differences of resulting therapy regimens compared to the pDST-derived treatments are highlighted by black boxes. Vertical bars indicate data for 15 drugs for each patient, i.e., from left to right, isoniazid (H), rifampin (R), rifabutin (Rb), ethambutol (E), pyrazinamide (Z), kanamycin (Km), amikacin (Am), capreomycin (Cm), ofloxacin (Ox), moxifloxacin (Mx), levofloxacin (Lx), prothionamide (Pt), para-aminosalicylic acid (Pa), cycloserine (Cs), terizidone (Tz), linezolid (Lz), amoxicillin-clavulanic acid (Ac), meropenem (Me), clofazimine (Cf), delamanid (De), and bedaquiline (Bq).

Three hundred sixty-seven pDST results for a total of 15 drugs served as the reference standard (Fig. 1). Xpert classified all 25 patients as having rifampin resistance, yet one isolate was phenotypically susceptible, resulting in an agreement of 96% (95% confidence interval [CI], 80 to 100%). LPA and pDST results agreed in 228 of 243 cases (94% [95% CI, 90 to 97%]). Three hundred forty of the 367 WGS-based drug resistance predictions (93% [95% CI, 89 to 95%]) were concordant with pDST (Fig. 1A and Table S2).

**FIG 2:**
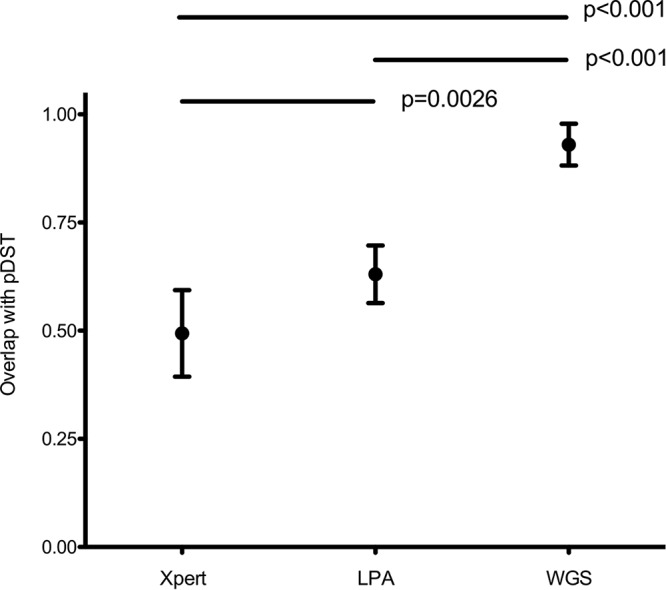
Average overlap of different regimens based on molecular DST assays compared with pDST results. Standard algorithm-derived treatment regimens based on results of Xpert, LPAs, and WGS (*x* axis) with their mean overlap to standard algorithm-derived treatment regimens based on pDST results (*y* axis). Mean overlaps (dots) are expressed with 95% confidence intervals (bars). *P* values assessing the differences between the mean overlaps between the treatment regimens are shown above.

There was a 49% (95% CI, 39 to 59%) average agreement in the number of antibiotics prescribed between the regimens based on Xpert results alone and those based on pDST (Fig. 2 and Table S3) (3). This increased to 68% (95% CI, 56 to 80%) if resistance to both ethambutol and pyrazinamide was also assumed based on the discovery of rifampin resistance. Making the equivalent assumption for LPAs increased the agreement from 63% (95% CI, 56 to 70%) to 87% (95% CI, 80 to 94%). The best agreement with pDST regimens was achieved with WGS (93% [95% CI, 88 to 98%]) (Fig. 2 and Table S3). Importantly, the WGS regimens did not feature any drugs to which resistance was found using pDST. In contrast, the 25 regimens that were designed using LPAs or Xpert contained 56/152 (37% [95% CI, 29 to 56]) and 77/150 (51% [95% CI, 43 to 60%]) drugs, respectively, for which pDST showed resistance (Table S4).

A more detailed analysis of drug categories revealed that the Xpert regimens involved an increased administration of group A, B, and D1 drugs compared with those for pDST (*P* < 0.001) (Table S5). Moreover, no D2 and D3 drugs were part of these regimens (*P* < 0.001). For the LPA regimens, only the increase in the number of D1 drugs was statistically significant. In contrast, the use of WGS resulted in a significant decrease in the use of D1 drugs because more ethambutol resistance was predicted (Table S5).

### Analysis of the discrepancies between different DST methods.

We determined the MICs for selected isolates and antibiotics to investigate the potential causes of the discrepancies observed with the different DST methods (Table S2).

### Rifampin and rifabutin.

**FIG 3:**
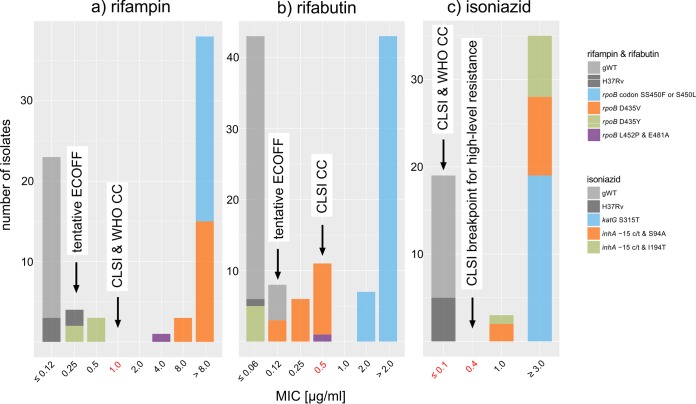
MIC distributions for rifampin, rifabutin, and isoniazid. (A and B) The CCs for rifampin and rifabutin were two dilutions higher than the tentative ECOFFs defined based on the pooled MIC data from this study and the literature (i.e., 1 versus 0.25 μg/ml for rifampin and 0.5 versus 0.12 μg/ml for rifabutin) (17). These distinctions did not make a difference for isolates with *rpoB* S450F or S450L mutations, which resulted in large MIC increases for both drugs. In contrast, the result of susceptible/resistant to rifampin by pDST for the *rpoB* D435Y isolate (11102-14), as well as the rifabutin results for the *rpoB* D435V and L452P/E481A isolates (12041-13 and 999-13), likely were breakpoint artifacts, as the isolates had elevated MICs compared to those of gWT isolates and the H37Rv laboratory strain. In contrast, the *rpoB* D435Y isolate appeared to be genuinely susceptible to rifabutin. However, lowering the CCs for both drugs to the ECOFFs would not necessarily ensure that isolates with elevated MICs always test resistant phenotypically. For example, because the MIC distribution of *rpoB* D435V (0.12 to 0.5 μg/ml) overlapped the gWT distribution of rifabutin, the normal variation in MIC testing would result in a poor reproducibility of pDST for this mutation. (C) WHO has only endorsed a single critical concentration for isoniazid, whereas CLSI has set an additional breakpoint that defines high-level resistance. Some treatment guidelines recommend the treatment of low-level resistant strains with a high dose of isoniazid (18). All mutant isolates were found to be resistant even at the second CLSI breakpoint, which was in accordance with our prediction based on WGS data (18). This would not have been apparent using the GenoType MTBDR*plus* assay, given that it only interrogates *inhA* promoter mutations, which typically result in low MICs, although this did not affect our interpretation of the assay, since we only relied on the WHO CCs (18).

One isolate (11102-14) with an *rpoB* D435Y mutation had a MIC for rifampin that was below the CC but above the tentative ECOFF defined in this study (tentative ECOFF of 0.25 μg/ml < *rpoB* mutant ECOFF of 0.5 μg/ml < CC of 1 μg/ml), which suggested that the susceptible pDST result represented a breakpoint artifact (Fig. 3A). This isolate also tested susceptible to rifabutin at the CC of 0.5 μg/ml (Fig. 3B). In this case, however, the result was likely valid, as its MIC (0.06 μg/ml) was even lower than the tentative ECOFF (0.12 μg/ml). In contrast, the susceptible pDST results with rifabutin for the D435Y and L452P/E481A isolates (12041-13 and 999-13) again were likely the result of breakpoint artifacts (17).

### Isoniazid and prothionamide.

All gWT isolates tested susceptible at the CLSI and WHO CC of 0.1 μg/ml. Conversely, all isolates with elevated MICs had known resistance mutations. Although not endorsed by WHO and not considered for our hypothetical regimens, CLSI has set 0.4 μg/ml as an additional breakpoint to define low-level resistance that can be treated with a high dose of isoniazid according to some recommendations (Fig. 3C) (18). Based on our WGS results, we were able to predict that all gNWT isolates were resistant even at this higher concentration (either because of the *katG* S315T mutation, which is known to confer predominantly high-level resistance, or because the isolates harbored both the *inhA* −15c/t promoter mutation and *inhA* coding changes [S94A or I194T] [18, 19]). It was not possible to predict the correct level of resistance for the *inhA* double mutants using MTBDR*plus*, given that this assay only interrogates promoter mutations (20).

For prothionamide, we observed only a single disagreement between our WGS predictions and those for pDST (21). Isolate 3758-14 originally tested susceptible despite a frameshift mutation in *ethA* (22). However, this discrepancy was likely a random error, since the isolate was found to have an elevated MIC compared with the CC (>25 μg/ml versus 2.5 μg/ml, respectively).

### Levofloxacin and moxifloxacin.

**FIG 4:**
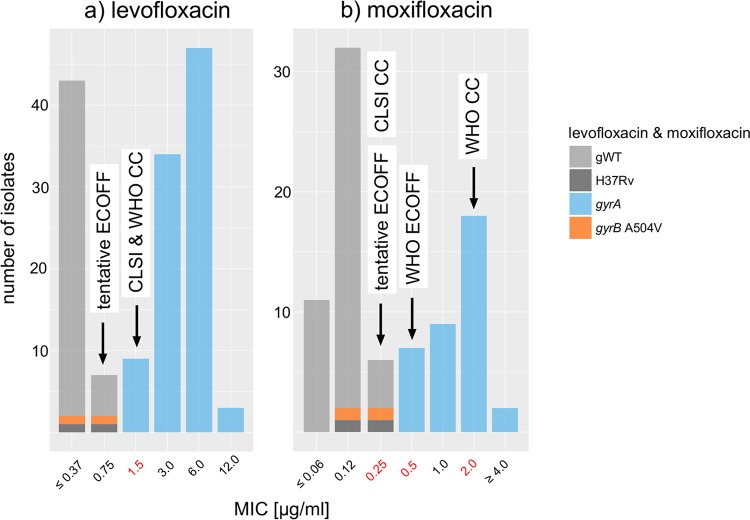
MIC distributions for levofloxacin and moxifloxacin. The pooled MIC data identified potential breakpoint artifacts for both agents. First, the CLSI and WHO critical concentrations for levofloxacin were one dilution higher than the tentative ECOFF defined in this study (1.5 versus 0.75 μg/ml) (11, 14). Second, the pooled data supported the current CLSI critical concentration (0.25 μg/ml) as the tentative ECOFF for moxifloxacin rather than the value set by WHO (0.5 μg/ml), which is designed as a surrogate for testing resistance to ofloxacin and levofloxacin (24). Moreover, WHO has acknowledged that the critical concentration at 2 μg/ml, which defines resistance to moxifloxacin, may be too high (24). Because two isolates with different genetic backgrounds shared the same *gyrB* A504V mutations, which is typically a signal of positive selection, these isolates were categorized as unclear. However, MIC testing revealed MICs that were equal to or below even the tentative ECOFFs for both fluoroquinolones, which was in line with allelic exchange experiments (59).

All seven isolates with known *gyrA* resistance mutations were resistant to levofloxacin at the CC of 1.5 μg/ml (23). However, a review of MIC data from the literature revealed a tentative ECOFF of 0.75 μg/ml, which resulted in the misclassification of 9 *gyrA* isolates from the literature (Fig. 4A).

WHO has set two CCs for moxifloxacin. The lower CC, at 0.5 μg/ml, is supposed to correspond to the ECOFF and is intended as a surrogate for ofloxacin and levofloxacin resistance (14, 24). However, our pooled MIC data suggested that the tentative ECOFF was actually 0.25 μg/ml, which was in agreement with the current CLSI guidelines (Fig. 4B) (11). All of our *gyrA* mutants were resistant at 2 μg/ml, the second WHO CC, which should define resistance to moxifloxacin itself (i.e., isolates with only slightly elevated MICs of 1 and 2 μg/ml are deemed to still be treatable with moxifloxacin). However, in light of the fact that WHO has already acknowledged that this CC may be too high and given that predicting the precise MIC based on genotypic data alone is challenging, we simply classified our isolates as gNWT (24).

### SLIDs.

**FIG 5:**
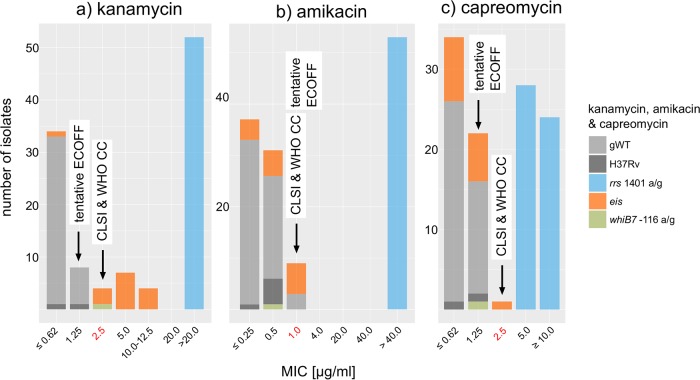
MIC distributions for kanamycin, amikacin, and capreomycin. The direct alteration of *rrs*, the shared target of kanamycin, amikacin, and capreomycin, via the A1401G mutation is known to confer unequivocal cross-resistance to all three drugs, which was in agreement with the pooled MIC data (60). In contrast, the current CCs for kanamycin were found to truncate the MIC distribution for isolates with *eis* and *whiB7* mutations (27). This meant that isolates with a MIC of 2.5 μg/ml were misclassified as susceptible despite the fact that these included mutations had been shown to result in elevated MICs using allelic exchange experiments (i.e., *eis* −37 g/t, *eis* −10 g/a, and *whiB7* −116 a/g) (25, 26). In contrast, neither *eis* nor *whiB7* mutations had a significant impact on the MICs of amikacin or capreomycin (based on previous data, the fact that the tentative ECOFF for capreomycin for our study was below the critical concentration was likely an artifact due to the small number of gWT isolates included in this study) (61).

The MIC distribution for isolates with known mutations in the resistance genes *eis* and *whiB7* ranged from 2.5 to 10 to 12.5 μg/ml and was truncated by the current CC of 2.5 μg/ml, whereas all gWT isolates had MICs of ≤0.125 μg/ml (25–27). Therefore, the two isolates with a MIC of 2.5 μg/ml (12471-13 and 11411-14) would have tested resistant if the CC was lowered to the tentative ECOFF of 1.25 μg/ml (Fig. 5A and Table S2). Moreover, we predict isolate 811-15, which had a known *whiB7* resistance mutation (−56 g/a), would retest resistant at 1.25 μg/ml (it tested susceptible at 2.5 μg/ml, and no MIC data were available for this isolate) (26). Two isolates had a previously unknown deletion of the upstream and coding regions of *eis*, which resulted in an invalid result with the MTBDR*sl* assay. The effect of this change on kanamycin resistance remains to be determined.

No discrepancies were observed for amikacin and capreomycin (28).

### Other antibiotics.

No discrepancies were found for streptomycin and pyrazinamide (29–33). For linezolid, isolate 9685-14 had a novel 23S mutation (*rrl* 906 g/a) that was observed in a susceptible isolate.

For the remaining antibiotics, we found evidence of false-susceptible pDST results. In the case of ethambutol, all 25 isolates were classified as gNWT but four tested susceptible (34–36). Up to five isolates, as opposed to two just phenotypically confirmed isolates, might have been cycloserine resistant given that the recently proposed tentative ECOFF of 20 μg/ml is below the CC of 30 μg/ml (37). Finally, up to six additional isolates could have been resistant to para-aminosalicylic acid based on the WGS data (see results in the supplemental material).

## DISCUSSION

We investigated how different genotypic DST assays influence the design of standardized algorithm-derived M/XDR-TB regimens. As expected, the accuracy of predicting resistance and, consequently, the ability to design appropriate treatment regimens correlated with the proportion of the genome analyzed. Moreover, we demonstrated that the pDST results were flawed in some cases.

Although LPAs have been endorsed by the WHO for the rapid molecular prediction of drug resistance of rifampin, isoniazid, fluoroquinolones, and SLIDs, Xpert is the most frequently used assay for initial routine molecular DST in many high-burden countries (6). Based on our results, it is a good test to rule in rifampin-resistant TB that can be used as surrogate marker for M/XDR-TB depending on the geographical region. However, it is paramount that these results are complemented with additional DST, since treatment regimens based only on an Xpert result would have led to the ineffective administration of approximately half of the drugs in this cohort of patients who were predominantly from eastern Europe. This will be different in other geographic settings, where the extent of drug resistance beyond rifampin and isoniazid is lower (38, 39).

The prediction of resistance to fluoroquinolones and SLIDs by LPAs was generally accurate for patients in this cohort. However, this test was also insufficient to construct appropriate M/XDR-TB regimens compared with pDST, especially in patients with XDR-TB. For example, almost all of the patients with M/XDR-TB from this cohort had strains that were resistant to ethambutol and pyrazinamide, which are not covered by the MTBDR*sl* 2.0. This was in line with results from a European study at 26 different centers in high-intermediate- and low-TB-burden countries that reported resistance to pyrazinamide and ethambutol in 59.7% and 59.3% of all patients with MDR-TB (94.4% and 81.8% of patients with XDR-TB), respectively (38, 39).

The M/XDR-TB treatment regimens based on WGS showed the highest agreement (93% [95% CI, 88 to 98%]) with those based on pDST. Unlike the other genotypic assays, WGS did not miss any phenotypically confirmed resistances but did predict resistance in some phenotypically susceptible isolates. This was partly due to the fact that we identified novel or poorly defined mutations that we could not interpret with regard to their impact on resistance development (e.g., mutations in *rrl* or *gyrB*; Table S2). Here, we adopted a conservative approach and assumed that these mutations conferred resistance, until disproved by another method, e.g., MIC determination of mutants derived from allelic exchange experiments and sequential patient-derived isolates that allow the interpretation of individual mutations and their effect on the drug resistance level in a particular phylogenetic strain background.

In other cases, problems with pDST played a role. The false-susceptible pDST results for ethambutol were likely due to the fact that some resistance mutations only result in slight MIC increases, which means that it can be difficult to distinguish the gWT strains from gNWT strains using pDST, unless secondary mutations increase the MICs even further (14, 40–42). The lack of reproducibility of pDST was also apparent for isolate 3758-14, which initially tested susceptible to prothionamide but became resistant upon retesting (Table S2).

Our results highlighted breakpoint artifacts (i.e., cases in which the current CCs were likely set above the tentative ECOFFs) as a major cause for systematic errors. In the absence of well-documented, high-quality evidence that isolates with elevated MICs can be treated with the standard or an elevated dose, the CCs for these drugs should be lowered to the tentative ECOFFs to avoid misdiagnosing isolates with elevated MICs as susceptible (12, 13). One possibility to gather such evidence would be to conduct a placebo-controlled study in which high-dose rifampin or rifabutin is used to treat low-level *rpoB* resistance mutations as part of a backbone M/XDR-TB regimen (43).

Importantly, we raised the possibility that breakpoint artifacts exist for six drugs that constitute the backbone of the treatment of drug-susceptible TB or MDR-TB (i.e., rifampin, levofloxacin, moxifloxacin, and kanamycin) in addition to less widely used drugs (i.e., rifabutin and cycloserine). The impact of this phenomenon depends on the geographic setting. For example, low-level resistance mutations in *rpoB* account for more than 10% of rifampin resistance in Bangladesh but are less frequent in other countries (44, 45). Problems related to kanamycin pDST likely are important in eastern Europe, where *eis* mutations are widespread among the dominant MDR-TB clones (46, 47).

This study was limited given that it was retrospective and only featured a small number of MDR and XDR patients from a single center, although the comparison between genotypic DST and pDST was strengthened by inclusion of MIC determinations of fully susceptible isolates from Sweden (*n* = 15). Our results did not provide direct evidence that treatment regimens based on different genotypic DST methods have an impact on clinical outcomes. Moreover, data from more laboratories including both drug-resistant and drug-susceptible isolates are required to set ECOFFs with confidence (16, 48). Nevertheless, the fact that potential breakpoint artifacts were found for so many key drugs underlines the urgent need for both CLSI and WHO to reexamine their CCs, which were set largely based on expert opinion using evidence that was not or was insufficiently documented, as opposed to modern and transparent principles pioneered by the European Committee on Antimicrobial Susceptibility Testing (EUCAST) (6, 12, 16). Importantly, this should include clear recommendations about how to proceed when discrepant results between genotypic assays and pDST are found (49). Ideally, these recommendations should consider MICs as well as clinical outcome data.

In conclusion, the strength of this study was that instead of merely calculating the concordance of genotypic DST results with those of pDST, as is customary for these assessments, we also compared the resulting regimens. In our view, this is more clinically meaningful, as TB is never treated with a single drug (in effect, we assessed the situation in settings that lack the laboratory infrastructure for pDST or, alternatively, the period while pDST is being carried out, but these results are not yet available). This is an important distinction, since the concordance of a genotypic DST assay with pDST can be deceptively high (96% [95% CI, 80 to 100%] for Xpert in our case), yet more than half of the drugs in the resulting regimens would still be prescribed inappropriately. Therefore, Xpert and LPA results should only be used to rule in resistance to WHO group A/B drugs and need to be complemented with further testing. WGS can provide important additional information on resistance to WHO group C/D drugs but cannot replace pDST completely either (e.g., pDST is still needed for novel mutations and to detect resistance caused by known resistance mutations that occur at frequencies below the detection limit of WGS [6]). Finally, the CCs need to be reevaluated to avoid systematic false-susceptible pDST results for a variety of first- and second-line drugs.

## MATERIALS AND METHODS

### Study population.

All patients (*n* = 25) with a diagnosis of M/XDR-TB admitted to the Medical Clinic of the Research Center Borstel (Germany) between March 2013 and March 2015 were included consecutively in the study.

### Microbiology, pDST, and MIC testing.

The primary detection, enrichment, DST, and MIC testing for the Germany isolates were done under routine conditions at the German National Reference Laboratory for Mycobacteria, Borstel. The following CCs, in micrograms per milliliter, were used for pDST with the Bactec 960 MGIT system using a critical proportion of 1% for all drugs, with the exception of pyrazinamide, for which 10% was employed: rifampin (1.0), rifabutin (0.5), isoniazid (0.1), prothionamide (2.5), ofloxacin (2.0), levofloxacin (1.5), moxifloxacin (0.5 and 2.0), kanamycin (2.5), amikacin (1.0), capreomycin (2.5), *para*-aminosalicylic acid (4.0), streptomycin (1.0), ethambutol (5.0), pyrazinamide (100.0), and linezolid (1.0) (11, 14). Cycloserine was tested using the proportion method on Löwenstein-Jensen medium using a CC of 30 μg/ml and a critical proportion of 1% (14).

The following concentrations, in micrograms per milliliter, were included for MGIT MIC testing for clinical isolates: rifampin (0.12, 0.25, 0.5, 1.0, 4.0, and 20.0), rifabutin (0.06, 0.12, 0.25, 0.5, 2.0, and 10.0), isoniazid (0.1, 0.4, 1.0, 3.0, and 10.0), prothionamide (0.62, 1.25, 2.5, 5.0, 10.0, and 25.0), levofloxacin (0.18, 0.37, 0.75, and 1.5), moxifloxacin (0.06, 0.12, 0.25, and 0.5), kanamycin (0.31, 0.62, 1.25, 2.5, 5.0, 12.5, and 25.0), amikacin (0.12, 0.25, 0.5, 1.0, 4.0, 20.0, and 40.0), capreomycin (0.31, 0.62, 1.25, 2.5, 5.0, 12.5, and 25.0), and para-aminosalicylic acid (0.5, 1.0, 2.0, and 4.0). The following concentrations ranges, in micrograms per milliliter, were tested in 2-fold dilutions for the M. tuberculosis H37Rv ATCC 27294 reference strain: rifampin (0.06 to 0.5), rifabutin (0.06 to 0.5), isoniazid (0.006 to 0.05), prothionamide (0.31 to 2.5), levofloxacin (0.09 to 1.5), moxifloxacin (0.06 to 0.5), kanamycin (0.31 to 2.5), amikacin (0.12 to 1), capreomycin (0.31 to 2.5), *para*-aminosalicylic acid (0.5 to 4), and linezolid (0.12 to 1).

### Molecular DSTs.

All baseline sputum specimens were analyzed with the Xpert assay according to the recommendations of the manufacturer. Genomic DNA extracted with cetyltrimethylammonium bromide from Löwenstein-Jensen cultures was used for the MTBDR*plus* 2.0 and MTBDR*sl* 2.0 LPAs as well as for WGS using a modified Illumina NexteraXT protocol and the MiSeq or NextSeq sequencer (20, 50–52). The detection of an *inhA* promoter variant with the MTBDR*plus* was used to infer prothionamide resistance (18). The raw data (fastq files) were submitted to the European Nucleotide Archive (see Table S2 in the supplemental material). Resulting reads were aligned to the M. tuberculosis H37Rv genome (GenBank accession no. NC_000962.3) using BWA-MEM (53). The GATK software package was utilized for base quality recalibration and alignment correction for possible PCR or insertion/deletion artifacts (54). Polymorphisms with a minimum of 10× coverage and 75% variant frequency were extracted and combined for all isolates using customized perl scripts. We focused our analysis on 33 resistance genes (Table S6) for which known polymorphisms that do not correlate with resistance (i.e., phylogenetic variants) were excluded (Table S7) (5, 55, 56).

WGS data were analyzed as follows (15). Isolates that did not have any mutations or only harbored neutral polymorphisms in drug resistance genes (Table S7) were classified as genotypically wild type and were assumed to be susceptible (gWT-S). Isolates with mutations known to result in MICs above the current CC that defines resistance [i.e., MICs > CC(R)] were classified as genotypically non-wild type and resistant (gNWT-R). Where two CCs have been set to define intermediate resistance (i.e., isolates that are treatable with an elevated dose of the drug), isolates with mutations that result in MICs within this range [i.e., CC(S) < MIC ≤ CC(R)] were gNWT intermediate (gNWT-I). gNWT susceptible (gNWT-S) was used to refer to isolates with mutations that confer elevated MICs below the lowest CC [i.e., ECOFF < MIC ≤ CC(S)]. Isolates with likely or known resistance mutations that do not necessarily result in MICs above the CC(S/R) (i.e., in the case of ethambutol and kanamycin) or that confer MIC increases above the CC(S) but not necessarily above the CC(R) were classified as simply gNWT. Mutations with no or insufficient evidence with regard to their effect on MICs were classified as unclear.

### Algorithm-derived treatment regimens.

We retrospectively designed treatment regimens based on the results obtained from each DST method (pDST, Xpert, LPAs, and WGS) using current MDR-TB treatment recommendations, as outlined in the supplemental material (3). To err on the side of caution, unclear and gNWT mutations from WGS were considered to be resistant. The 367 initial pDST results served as a reference standard for all comparisons (15 drugs for 25 patients with eight missing results, which could not be conducted because of biosafety concerns).

### Statistics.

Concordance between each diagnostic test result with phenotypic DST was scored for every individual on a scale from 0 to 1, with 0 representing no concordance and 1 perfect concordance for each individual test result. The same approach was used to assess the overlap between the different treatment regimens for each individual regimen. Differences in scores were evaluated using the Mann-Whitney U test. The overlap between different diagnostic methods and the agreement between the different treatment regimens were evaluated using the differences in proportions where each drug from a given group was considered independently. Graphs were created and statistics calculated using STATA version 14 (STATA Corp., Texas, USA) and Prism version 5 (GraphPad Software Inc., La Jolla, California, USA). *P* values below 0.05 were considered significant.

### Determining tentative ECOFFs.

We set tentative ECOFFs by visual inspection for a variety of antibiotics (statistical methods could not be used given the MIC data did not meet the minimum requirements specified by EUCAST to set ECOFFs [48]). For this purpose, we pooled the MICs from the German patient cohort with MICs from a Swedish collection (see the supplemental material) and the literature wherever the individual concentrations and concentration ranges were sufficiently similar (17, 19, 27, 57, 58). As shown in Table S8, we had to truncate some of the distributions for this purpose. For Kambli et al. we excluded one isolate for which the genetic basis of the elevated MICs was not clear (27). We did not display the MICs for *gyrB* mutations from Nosova et al. given the mutations differed from the *gyrB* A504V mutation observed in our study (57). We only included MIC data for *rpoB* mutations from Berrada et al. that also occurred in the German isolates (17).

### Ethics.

The ethics committee of the University of Lübeck, Germany, approved the study (15-195A). Approval for whole-genome sequencing and analysis of the isolates from Sweden was granted by the UK National Research Ethics Service (12/EE/0439) and the Cambridge University Hospitals NHS Foundation Trust R&D Department (A092685).

## Supplementary Material

Supplemental material

## References

[1] World Health Organization. 2016 Global tuberculosis report. World Health Organization, Geneva, Switzerland http://apps.who.int/iris/bitstream/10665/250441/1/9789241565394-eng.pdf?ua=1.

[2] Lange C, Abubakar I, Alffenaar JWC, Bothamley G, Caminero Ja Carvalho ACC, Chang KC, Codecasa L, Correia A, Crudu V, Davies P, Dedicoat M, Drobniewski F, Duarte R, Ehlers C, Erkens C, Goletti D, Günther G, Ibraim E, Kampmann B, Kuksa L, De Lange W, Van Leth F, Van Lunzen J, Matteelli A, Menzies D, Monedero I, Richter E, Rüsch-Gerdes S, Sandgren A, Scardigli A, Skrahina A, Tortoli E, Volchenkov G, Wagner D, Van Der Werf MJ, Williams B, Yew WW, Zellweger JP, Cirillo DM 2014 Management of patients with multidrugresistant/extensively drug-resistant tuberculosis in Europe: a TBNET consensus statement. Eur Respir J 44:23–63. doi:10.1183/09031936.00188313. 24659544 PMC4076529

[3] Horsburgh CR Jr, Barry CE III, Lange C 2015 Treatment of tuberculosis. N Engl J Med 373:2149–2160. doi:10.1056/NEJMra1413919. 26605929

[4] Köser CU, Ellington MJ, Cartwright EJ, Gillespie SH, Brown NM, Farrington M, Holden MT, Dougan G, Bentley SD, Parkhill J, Peacock SJ 2012 Routine use of microbial whole genome sequencing in diagnostic and public health microbiology. PLoS Pathog 8:e1002824. doi:10.1371/journal.ppat.1002824. 22876174 PMC3410874

[5] Köser CU, Bryant JM, Becq J, Török ME, Ellington MJ, Marti-Renom MA, Carmichael AJ, Parkhill J, Smith GP, Peacock SJ 2013 Whole-genome sequencing for rapid susceptibility testing of *M. tuberculosis*. N Engl J Med 369:290–292. doi:10.1056/NEJMc1215305. 23863072 PMC3836233

[6] Schön T, Miotto P, Köser CU, Viveiros M, Böttger E, Cambau E 2017 *Mycobacterium tuberculosis* drug-resistance testing: challenges, recent developments and perspectives. Clin Microbiol Infect 23:154–160. doi:10.1016/j.cmi.2016.10.022. 27810467

[7] Boehme CC, Nabeta P, Hillemann D 2010 Rapid molecular detection of tuberculosis and rifampin resistance. N Engl J Med 363:1005–1015. doi:10.1056/NEJMoa0907847. 20825313 PMC2947799

[8] Walker TM, Kohl TA, Omar SV, Hedge J, Del Ojo Elias C, Bradley P, Iqbal Z, Feuerriegel S, Niehaus KE, Wilson DJ, Clifton DA, Kapatai G, Ip CL, Bowden R, Drobniewski FA, Allix-Beguec C, Gaudin C, Parkhill J, Diel R, Supply P, Crook DW, Smith EG, Walker AS, Ismail N, Niemann S, Peto TE, Modernizing Medical Microbiology Informatics Group. 2015 Whole-genome sequencing for prediction of *Mycobacterium tuberculosis* drug susceptibility and resistance: a retrospective cohort study. Lancet Infect Dis 15:1193–1202. doi:10.1016/S1473-3099(15)00062-6. 26116186 PMC4579482

[9] McNerney R, Clark TG, Campino S, Rodrigues C, Dolinger D, Smith L, Cabibbe AM, Dheda K, Schito M 2017 Removing the bottleneck in whole genome sequencing of *Mycobacterium tuberculosis* for rapid drug resistance analysis: a call to action. Int J Infect Dis 56:130–135. doi:10.1016/j.ijid.2016.11.422. 27986491

[10] Walker TM, Merker M, Kohl TA, Crook DW, Niemann S, Peto TEA 2016 Whole genome sequencing for M/XDR tuberculosis surveillance and for resistance testing. Clin Microbiol Infect 23:161–166. doi:10.1016/j.cmi.2016.10.014. 27789378

[11] Clinical and Laboratory Standards Institute. 2011 Susceptibility testing of mycobacteria, nocardiae, and other aerobic actinomycetes, 2nd ed Approved standard CLSI document M24-A2. Clinical and Laboratory Standards Institute, Wayne, PA. 31339680

[12] Ängeby K, Juréen P, Kahlmeter G, Hoffner SE, Schön T 2012 Challenging a dogma: antimicrobial susceptibility testing breakpoints for *Mycobacterium tuberculosis*. Bull World Health Organ 90:693–698. doi:10.2471/BLT.11.096644. 22984314 PMC3442398

[13] Schön T, Juréen P, Chryssanthou E, Giske CG, Kahlmeter G, Hoffner S, Ängeby K 2013 Rifampicin-resistant and rifabutin-susceptible *Mycobacterium tuberculosis* strains: a breakpoint artefact? J Antimicrob Chemother 68:2074–2077. doi:10.1093/jac/dkt150. 23633684

[14] World Health Organization. 2014 Companion handbook to the WHO guidelines for the programmatic management of drug-resistant tuberculosis. World Health Organization, Geneva, Switzerland http://appswhoint/iris/bitstream/10665/130918/1/9789241548809_engpdf?ua=1&ua=1. 25320836

[15] Ellington MJ, Ekelund O, Aarestrup FM, Canton R, Doumith M, Giske C, Grundman H, Hasman H, Holden MT, Hopkins KL, Iredell J, Kahlmeter G, Koser CU, MacGowan A, Mevius D, Mulvey M, Naas T, Peto T, Rolain JM, Samuelsen O, Woodford N 2017 The role of whole genome sequencing in antimicrobial susceptibility testing of bacteria: report from the EUCAST Subcommittee. Clin Microbiol Infect 23:2–22. doi:10.1016/j.cmi.2016.11.012. 27890457

[16] Kahlmeter G 2015 The 2014 Garrod lecture: EUCAST–are we heading towards international agreement? J Antimicrob Chemother 70:2427–2439. doi:10.1093/jac/dkv145. 26089441

[17] Berrada ZL, Lin SY, Rodwell TC, Nguyen D, Schecter GF, Pham L, Janda JM, Elmaraachli W, Catanzaro A, Desmond E 2016 Rifabutin and rifampin resistance levels and associated *rpoB* mutations in clinical isolates of *Mycobacterium tuberculosis* complex. Diagn Microbiol Infect Dis 85:177–181. doi:10.1016/j.diagmicrobio.2016.01.019. 27036978 PMC4873381

[18] Domínguez J, Boettger EC, Cirillo D, Cobelens F, Eisenach KD, Gagneux S, Hillemann D, Horsburgh R, Molina-Moya B, Niemann S, Tortoli E, Whitelaw A, Lange C, TBNET, RESIST-TB Networks. 2016 Clinical implications of molecular drug resistance testing for *Mycobacterium tuberculosis*: a TBNET/RESIST-TB consensus statement. Int J Tuberc Lung Dis 20:24–42. doi:10.5588/ijtld.15.0221. 26688526

[19] Machado D, Perdigão J, Ramos J, Couto I, Portugal I, Ritter C, Boettger EC, Viveiros M 2013 High-level resistance to isoniazid and ethionamide in multidrug-resistant *Mycobacterium tuberculosis* of the Lisboa family is associated with *inhA* double mutations. J Antimicrob Chemother 68:1728–1732. doi:10.1093/jac/dkt090. 23539241

[20] Nathavitharana RR, Hillemann D, Schumacher SG, Schlueter B, Ismail N, Omar SV, Sikhondze W, Havumaki J, Valli E, Boehme C, Denkinger CM 2016 Multicenter noninferiority evaluation of Hain GenoType MTBDR*plus* version 2 and Nipro NTM+MDRTB line probe assays for detection of rifampin and isoniazid resistance. J Clin Microbiol 54:1624–1630. doi:10.1128/JCM.00251-16. 27076658 PMC4879293

[21] Ando H, Miyoshi-Akiyama T, Watanabe S, Kirikae T 2014 A silent mutation in *mabA* confers isoniazid resistance on *Mycobacterium tuberculosis*. Mol Microbiol 91:538–547. doi:10.1111/mmi.12476. 24354762

[22] Dover LG, Alahari A, Gratraud P, Gomes JM, Bhowruth V, Reynolds RC, Besra GS, Kremer L 2007 EthA, a common activator of thiocarbamide-containing drugs acting on different mycobacterial targets. Antimicrob Agents Chemother 51:1055–1063. doi:10.1128/AAC.01063-06. 17220416 PMC1803108

[23] Maruri F, Sterling TR, Kaiga AW, Blackman A, van der Heijden YF, Mayer C, Cambau E, Aubry A 2012 A systematic review of gyrase mutations associated with fluoroquinolone-resistant *Mycobacterium tuberculosis* and a proposed gyrase numbering system. J Antimicrob Chemother 67:819–831. doi:10.1093/jac/dkr566. 22279180 PMC3299416

[24] Zignol M, Dean AS, Alikhanova N, Andres S, Cabibbe AM, Cirillo DM, Dadu A, Dreyer A, Driesen M, Gilpin C, Hasan R, Hasan Z, Hoffner S, Husain A, Hussain A, Ismail N, Kamal M, Mansjo M, Mvusi L, Niemann S, Omar SV, Qadeer E, Rigouts L, Ruesch-Gerdes S, Schito M, Seyfaddinova M, Skrahina A, Tahseen S, Wells WA, Mukadi YD, Kimerling M, Floyd K, Weyer K, Raviglione MC 2016 Population-based resistance of *Mycobacterium tuberculosis* isolates to pyrazinamide and fluoroquinolones: results from a multicountry surveillance project. Lancet Infect Dis 16:1185–1192. doi:10.1016/S1473-3099(16)30190-6. 27397590 PMC5030278

[25] Zaunbrecher MA, Sikes RD Jr, Metchock B, Shinnick TM, Posey JE 2009 Overexpression of the chromosomally encoded aminoglycoside acetyltransferase *eis* confers kanamycin resistance in *Mycobacterium tuberculosis*. Proc Natl Acad Sci U S A 106:20004–20009. doi:10.1073/pnas.0907925106. 19906990 PMC2785282

[26] Reeves AZ, Campbell PJ, Sultana R, Malik S, Murray M, Plikaytis BB, Shinnick TM, Posey JE 2013 Aminoglycoside cross-resistance in *Mycobacterium tuberculosis* due to mutations in the 5′ untranslated region of *whiB7*. Antimicrob Agents Chemother 57:1857–1865. doi:10.1128/AAC.02191-12. 23380727 PMC3623337

[27] Kambli P, Ajbani K, Nikam C, Sadani M, Shetty A, Udwadia Z, Georghiou SB, Rodwell TC, Catanzaro A, Rodrigues C 2016 Corrigendum to “Correlating *rrs* and *eis* promoter mutations in clinical isolates of *Mycobacterium tuberculosis* with phenotypic susceptibility levels to the second-line injectables” [Int J Mycobacteriol 5(1) 2016 1–6]. Int J Mycobacteriol 5:370–372. doi:10.1016/j.ijmyco.2016.06.009. 26927983 PMC4863938

[28] Maus CE, Plikaytis BB, Shinnick TM 2005 Mutation of *tlyA* confers capreomycin resistance in *Mycobacterium tuberculosis*. Antimicrob Agents Chemother 49:571–577. doi:10.1128/AAC.49.2.571-577.2005. 15673735 PMC547314

[29] Finken M, Kirschner P, Meier A, Wrede A, Böttger E 1993 Molecular basis of streptomycin resistance in *Mycobacterium tuberculosis*: alterations of the ribosomal protein S12 gene and point mutations within a functional 16S ribosomal RNA pseudoknot. Mol Microbiol 9:1239–1246. doi:10.1111/j.1365-2958.1993.tb01253.x. 7934937

[30] Dymova MA, Cherednichenko AG, Alkhovik OI, Khrapov EA, Petrenko TI, Filipenko ML 2014 Characterization of extensively drug-resistant *Mycobacterium tuberculosis* isolates circulating in Siberia. BMC Infect Dis 14:478. doi:10.1186/1471-2334-14-478. 25186134 PMC4161839

[31] Miotto P, Cabibbe AM, Feuerriegel S, Casali N, Drobniewski F, Rodionova Y, Bakonyte D, Stakenas P, Pimkina E, Augustynowicz-Kopec E, Degano M, Ambrosi A, Hoffner S, Mansjo M, Werngren J, Rüsch-Gerdes S, Niemann S, Cirillo DM 2014 *Mycobacterium tuberculosis* pyrazinamide resistance determinants: a multicenter study. mBio 5:e01819-14. doi:10.1128/mBio.01819-14. 25336456 PMC4212837

[32] Tekwu EM, Sidze LK, Assam JP, Tedom JC, Tchatchouang S, Makafe GG, Wetewale AL, Kuaban C, Eyangoh S, Ntoumi F, Beng VN, Frank M 2014 Sequence analysis for detection of drug resistance in *Mycobacterium tuberculosis* complex isolates from the Central Region of Cameroon. BMC Microbiol 14:113. doi:10.1186/1471-2180-14-113. 24884632 PMC4017682

[33] Ramirez-Busby SM, Valafar F 2015 Systematic review of mutations in pyrazinamidase associated with pyrazinamide resistance in *Mycobacterium tuberculosis* clinical isolates. Antimicrob Agents Chemother 59:5267–5277. doi:10.1128/AAC.00204-15. 26077261 PMC4538510

[34] Campbell PJ, Morlock GP, Sikes RD, Dalton TL, Metchock B, Starks AM, Hooks DP, Cowan LS, Plikaytis BB, Posey JE 2011 Molecular detection of mutations associated with first- and second-line drug resistance compared with conventional drug susceptibility testing of *Mycobacterium tuberculosis*. Antimicrob Agents Chemother 55:2032–2041. doi:10.1128/AAC.01550-10. 21300839 PMC3088277

[35] Cui Z, Li Y, Cheng S, Yang H, Lu J, Hu Z, Ge B 2014 Mutations in the *embC-embA* intergenic region contribute to *Mycobacterium tuberculosis* resistance to ethambutol. Antimicrob Agents Chemother 58:6837–6843. doi:10.1128/AAC.03285-14. 25182646 PMC4249443

[36] Nebenzahl-Guimaraes H, Jacobson KR, Farhat MR, Murray MB 2014 Systematic review of allelic exchange experiments aimed at identifying mutations that confer drug resistance in *Mycobacterium tuberculosis*. J Antimicrob Chemother 69:331–342. doi:10.1093/jac/dkt358. 24055765 PMC3886931

[37] Desjardins CA, Cohen KA, Munsamy V, Abeel T, Maharaj K, Walker BJ, Shea TP, Almeida DV, Manson AL, Salazar A, Padayatchi N, O'Donnell MR, Mlisana KP, Wortman J, Birren BW, Grosset J, Earl AM, Pym AS 2016 Genomic and functional analyses of *Mycobacterium tuberculosis* strains implicate *ald* in D-cycloserine resistance. Nat Genet 48:544–551. doi:10.1038/ng.3548. 27064254 PMC4848111

[38] Günther G, Leth FV, Alexandru S, Altet N, Avsar K, Bang D, Barbuta R, Bothamley G, Ciobanu A, Crudu V, Danilovits M, Dedicoat M, Duarte R, Gualano G, Kunst H, Lange WD, Leimane V, Magis-Escurra C, McLaughlin A-M, Muylle I, Polcová V, Pontali E, Popa C, Rumetshofer R, Skrahina A, Solodovnikova V, Spinu V, Tiberi S, Viiklepp P, Lange C 2015 Multidrug-resistant tuberculosis in Europe, 2010–2011. Emerg Infect Dis 21:2010–2011. 10.3201/eid2103.141343 PMC4344280 25693485

[39] Günther G, van Leth F, Altet N, Dedicoat M, Duarte R, Gualano G, Kunst H, Muylle I, Spinu V, Tiberi S, Viiklepp P, Lange C, TBNET. 2015 Beyond multidrug-resistant tuberculosis in Europe: a TBNET study. Int J Tuberc Lung Dis 19:1524–1527. 26614196 10.5588/ijtld.15.0274

[40] Böttger EC 2011 The ins and outs of Mycobacterium tuberculosis drug susceptibility testing. Clin Microbiol Infect 17:1128–1134. doi:10.1111/j.1469-0691.2011.03551.x. 21631641

[41] Safi H, Lingaraju S, Amin A, Kim S, Jones M, Holmes M, McNeil M, Peterson SN, Chatterjee D, Fleischmann R, Alland D 2013 Evolution of high-level ethambutol-resistant tuberculosis through interacting mutations in decaprenylphosphoryl-β-D-arabinose biosynthetic and utilization pathway genes. Nat Genet 45:1190–1197. doi:10.1038/ng.2743. 23995136 PMC6103293

[42] Yakrus MA, Driscoll J, McAlister A, Sikes D, Hartline D, Metchock B, Starks AM 2016 Molecular and growth-based drug susceptibility testing of *Mycobacterium tuberculosis* complex for ethambutol resistance in the United States. Tuberc Res Treat 2016:3404860. 27375902 10.1155/2016/3404860 PMC4916310

[43] Sirgel FA, Warren RM, Böttger EC, Klopper M, Victor TC, van Helden PD 2013 The rationale for using rifabutin in the treatment of MDR and XDR tuberculosis outbreaks. PLoS One 8:e59414. doi:10.1371/journal.pone.0059414. 23527189 PMC3602005

[44] Van Deun A, Aung KJ, Bola V, Lebeke R, Hossain MA, de Rijk WB, Rigouts L, Gumusboga A, Torrea G, de Jong BC 2013 Rifampin drug resistance tests for tuberculosis: challenging the gold standard. J Clin Microbiol 51:2633–2640. doi:10.1128/JCM.00553-13. 23761144 PMC3719626

[45] Gonzalo X, Claxton P, Brown T, Montgomery L, Fitzgibbon M, Laurenson I, Drobniewski F 2017 True rifampicin resistance missed by the MGIT: prevalence of this pheno/genotype in the UK and Ireland after 18 month surveillance. Clin Microbiol Infect 23:260–263. doi:10.1016/j.cmi.2016.11.015. 27903459

[46] Casali N, Nikolayevskyy V, Balabanova Y, Harris SR, Ignatyeva O, Kontsevaya I, Corander J, Bryant J, Parkhill J, Nejentsev S, Horstmann RD, Brown T, Drobniewski F 2014 Evolution and transmission of drug-resistant tuberculosis in a Russian population. Nat Genet 46:279–286. doi:10.1038/ng.2878. 24464101 PMC3939361

[47] Merker M, Blin C, Mona S, Duforet-Frebourg N, Lecher S, Willery E, Blum MG, Rüsch-Gerdes S, Mokrousov I, Aleksic E, Allix-Beguec C, Antierens A, Augustynowicz-Kopec E, Ballif M, Barletta F, Beck HP, Barry CE III, Bonnet M, Borroni E, Campos-Herrero I, Cirillo D, Cox H, Crowe S, Crudu V, Diel R, Drobniewski F, Fauville-Dufaux M, Gagneux S, Ghebremichael S, Hanekom M, Hoffner S, Jiao WW, Kalon S, Kohl TA, Kontsevaya I, Lillebaek T, Maeda S, Nikolayevskyy V, Rasmussen M, Rastogi N, Samper S, Sanchez-Padilla E, Savic B, Shamputa IC, Shen A, Sng LH, Stakenas P, Toit K, Varaine F, Vukovic D, Wahl C, Warren R, Supply P, Niemann S, Wirth T 2015 Evolutionary history and global spread of the *Mycobacterium tuberculosis* Beijing lineage. Nat Genet 47:242–249. doi:10.1038/ng.3195. 25599400 PMC11044984

[48] European Committee for Antimicrobial Susceptibility Testing. 2017 EUCAST subcommittee on MIC distributions and epidemiological cut-off values (ECOFFs). Discussion document, version 3. http://www.eucast.org/organization/subcommittees/mic_distributions_and_ecoffs/.

[49] Hofmann-Thiel S, Hoffmann H, Hillemann D, Rigouts L, Van Deun A, Kranzer K 2017 How should discordance between molecular and growth-based assays for rifampicin resistance be investigated? Int J Tuberc Lung Dis 21:721–726. doi:10.5588/ijtld.17.0140. 28633695

[50] van Embden JD, Cave MD, Crawford JT, Dale JW, Eisenach KD, Gicquel B, Hermans P, Martin C, McAdam R, Shinnick TM, Small PM 1993 Strain identification of *Mycobacterium tuberculosis* by DNA fingerprinting: recommendations for a standardized methodology. J Clin Microbiol 31:406–409. 8381814 10.1128/jcm.31.2.406-409.1993 PMC262774

[51] Baym M, Kryazhimskiy S, Lieberman TD, Chung H, Desai MM, Kishony R 2015 Inexpensive multiplexed library preparation for megabase-sized genomes. PLoS One 10:e0128036. doi:10.1371/journal.pone.0128036. 26000737 PMC4441430

[52] Tagliani E, Cabibbe AM, Miotto P, Borroni E, Toro JC, Mansjo M, Hoffner S, Hillemann D, Zalutskaya A, Skrahina A, Cirillo DM 2015 Diagnostic performance of the new version of GenoType MTBDR*sl* (V2.0) assay for detection of resistance to fluoroquinolones and second line injectable drugs: a multicenter study. J Clin Microbiol 53:2961–2969. doi:10.1128/JCM.01257-15. 26179309 PMC4540937

[53] Li H, Durbin R 2009 Fast and accurate short read alignment with Burrows-Wheeler transform. Bioinformatics 25:1754–1760. doi:10.1093/bioinformatics/btp324. 19451168 PMC2705234

[54] McKenna A, Hanna M, Banks E, Sivachenko A, Cibulskis K, Kernytsky A, Garimella K, Altshuler D, Gabriel S, Daly M, DePristo MA 2010 The Genome Analysis Toolkit: a MapReduce framework for analyzing next-generation DNA sequencing data. Genome Res 20:1297–1303. doi:10.1101/gr.107524.110. 20644199 PMC2928508

[55] Köser CU, Feuerriegel S, Summers DK, Archer JA, Niemann S 2012 Importance of the genetic diversity within the *Mycobacterium tuberculosis* complex for the development of novel antibiotics and diagnostic tests of drug resistance. Antimicrob Agents Chemother 56:6080–6087. doi:10.1128/AAC.01641-12. 23006760 PMC3497208

[56] Feuerriegel S, Köser CU, Niemann S 2014 Phylogenetic polymorphisms in antibiotic resistance genes of the *Mycobacterium tuberculosis* complex. J Antimicrob Chemother 69:1205–1210. doi:10.1093/jac/dkt535. 24458512

[57] Nosova EY, Bukatina AA, Isaeva YD, Makarova MV, Galkina KY, Moroz AM 2013 Analysis of mutations in the *gyrA* and *gyrB* genes and their association with the resistance of *Mycobacterium tuberculosis* to levofloxacin, moxifloxacin and gatifloxacin. J Med Microbiol 62:108–113. doi:10.1099/jmm.0.046821-0. 23019190

[58] Kambli P, Ajbani K, Nikam C, Khillari A, Shetty A, Udwadia Z, Georghiou SB, Rodwell TC, Catanzaro A, Rodrigues C 2015 Determination of MICs of levofloxacin for *Mycobacterium tuberculosis* with *gyrA* mutations. Int J Tuberc Lung Dis 19:1227–1229. doi:10.5588/ijtld.14.0277. 26459538 PMC4607084

[59] Malik S, Willby M, Sikes D, Tsodikov OV, Posey JE 2012 New insights into fluoroquinolone resistance in *Mycobacterium tuberculosis*: functional genetic analysis of *gyrA* and *gyrB* mutations. PLoS One 7:e39754. doi:10.1371/journal.pone.0039754. 22761889 PMC3386181

[60] Reeves AZ, Campbell PJ, Willby MJ, Posey JE 2015 Disparities in capreomycin resistance levels associated with the *rrs* A1401G mutation in clinical isolates of *Mycobacterium tuberculosis*. Antimicrob Agents Chemother 59:444–449. doi:10.1128/AAC.04438-14. 25385119 PMC4291384

[61] Rodrigues C, Jani J, Shenai S, Thakkar P, Siddiqi S, Mehta A 2008 Drug susceptibility testing of *Mycobacterium tuberculosis* against second-line drugs using the Bactec MGIT 960 system. Int J Tuberc Lung Dis 12:1449–1455. 19017456

