# Supplementary material for: What Is Resistance? Impact of Phenotypic versus Molecular Drug Resistance Testing on Therapy for Multi- and Extensively Drug-Resistant Tuberculosis
Source: Antimicrob Agents Chemother. 2018 Jan 25;62(2):e01550-17. doi: 10.1128/AAC.01550-17 (PMC5786814; doi:10.1128/AAC.01550-17)
Supplement: Supplemental material [file supp_62_2_e01550-17__index.html]

Supplemental material 

# What Is Resistance? Impact of Phenotypic versus Molecular Drug Resistance Testing on Therapy for Multi- and Extensively Drug-Resistant Tuberculosis

## Supplemental material

- Supplemental file 1 -

  Supplemental text and Tables S1 and S3 to S7

  PDF, 539K
- Supplemental file 2 -

  Table S2

  XLSX, 30K
- Supplemental file 3 -

  Table S8

  XLSX, 45K
