# Supplementary material for: What Is Resistance? Impact of Phenotypic versus Molecular Drug Resistance Testing on Therapy for Multi- and Extensively Drug-Resistant Tuberculosis
Source: Antimicrob Agents Chemother. 2018 Jan 25;62(2):e01550-17. doi: 10.1128/AAC.01550-17 (PMC5786814; doi:10.1128/AAC.01550-17)
Supplement: Supplemental material [file AAC.01550-17_zac002186843s1.pdf]

SUPPLEMENTARY METHODS

Treatment algorithm based on Horsburgh et al.[1]

- Use a stepwise approach to design a MDR-TB regimen.
- The regimen should include a combination of ≥4 drugs in MDR-TB (a combination of ≥5 drugs in XDR-TB) likely to be active against *M. tuberculosis*.
- Recommended total duration of the treatment is currently 20 months.

Step 1

In case of rifabutin susceptibility (and rifampicin resistance) use rifabutin in the drug regimen. Due to lack of data, it cannot be recommended at present to count rifabutin among the ≥4 active drugs in the MDR-TB regimen (≥5 drugs in XDR-TB).

Comments

- Rifabutin should be part of a MDR-TB therapy if *M. tuberculosis* is in vitro susceptible despite rifampicin resistance (usually related to a D516mut mutation in the *rpoB* gene). Monitor for hepatotoxicity.
- Dose adjustments may be necessary when dosing with interacting drugs (e.g. ART).
- Due to lack of data it is currently not recommended to count this drug among the number of drugs used for the treatment of MDR-TB or shorten the duration of therapy.

Step 2

In case of a low-level isoniazid drug resistance use high dose isoniazid in the drug regimen.

Comments

- In the absence of a *katG* S315T mutation, *inhA* promoter mutations (8A/C, 15T, 16G) confer low-level isoniazid resistance (MIC < 1 μg/ml) and treatment with isoniazid 16-20 mg/kg should be considered.
- Monitor for hepatotoxicity.

- Give with pyridoxine.
- Due to lack of data, it cannot be recommended at present to count high dose isoniazid among the ≥4 active drugs in the MDR-TB regimen (≥5 drugs in XDR-TB) or shorten the duration of therapy.
- With *inhA* promotor mutation ethionamide or prothionamide should not be included in the regimen, as *inhA* promotor mutations usually lead to thioamide drug resistance.

<u>Step 3</u>

Unless drug resistance to a later generation fluoroquinolone is suggested by genotypic of phenotypic drug susceptibility testing use moxifloxacin or levofloxacin in the drug regimen.

Comments

- Fluoroquinolones are probably the most effective 2nd line antituberculosis drugs available at present.
- QTc interval prolongation may be potentiated with other drugs. Close monitoring recommended when used with other drugs that prolong the QTc interval. Concurrent use of moxifloxacin with bedaquiline or delamanid not recommended.

<u>Step 4</u>

In case of later generation fluoroquinolone drug resistance, use bedaquiline in the drug regimen.

Comments

Bedaquiline is approved for the treatment of MDR-TB by the FDA and EMA for use as part of an appropriate combination regimen when an effective treatment regimen is unavailable because of resistance or intolerability to other medications. QTc interval prolongation may be potentiated with other drugs. Close monitoring recommended when used with other drugs that prolong the QTc interval. Concurrent use with delamanid or moxifloxacin not recommended.

Step 5

Unless drug resistance to a second line injectable drug (SLID, amikacin, capreomycin or kanamycin) is suggested by genotypic or phenotypic drug susceptibility testing use a SLID as part of the drug regimen.

Comments

If possible, a SLID should be included during the first 8 months of the treatment.

Capreomycin is preferred for reasons of drug-toxicity in long-term treatment. Patients should have a CVC catheter with subcutaneous reservoir implanted for daily intravenous therapy. Monitor renal function, electrolytes and audiology exam.

Step 6

Unless drug resistance to ethionamide/prothionamide, cycloserine/terizidone, PAS or linezolid is suggested by genotypic or phenotypic drug susceptibility testing, a combination of up to 3 of these drugs is often used.

Comments

- Prothionamide or ethionamide are often not tolerated in combination with PAS. Monitor liver and thyroid function. Give with pyridoxine.
- Terizidone is less toxic than cycloserine and is the fusion product of two molecules of cycloserine and one molecule of terephtalaldehyde. Monitor mental status. Give with pyridoxine.
- PAS is often not tolerated in combination with prothionamide or ethionamide.
- Severe adverse events are frequent in long-term therapy with linezolid, close monitoring of blood count and awareness of peripheral neuropathy is mandatory

Step 7

If a sufficient number of likely active drugs cannot be achieved in the regimen, add meropenem/amoxicillin-clavulanate and/or clofazimine and/or delamanid

Comments

- For the treatment of XDR-TB with meropenem/amoxicillin-clavulanate long term intravenous access recommended.
- When treatment with clofazimine causes severe skin discoloration dose reduction to five times a week may be justified. Monitor QTc interval.
- Delamanid is approved for the treatment of MDR-TB by the EMA for use as part of an appropriate combination regimen when an effective treatment regimen is unavailable because of resistance or intolerability to other medications. QTc interval prolongation may be potentiated with other drugs. Close monitoring recommended when used with other drugs that prolong the QTc interval. Concurrent use with bedaquiline or moxifloxacin not recommended.

Step 8

Pyrazinamide and/or ethambutol should be added to the initial treatment regimen, but the drugs should not be counted among the ≥4 active drugs in the MDR-TB regimen (≥5 drugs in XDR-TB regimen). Treatment with pyrazinamide and/or ethambutol should be discontinued, if results from phenotypic or genotypic drug susceptibility testing suggest resistance to either drug.

Comments

Phenotypic drug resistance to pyrazinamide and ethambutol is very common in M/XDR-TB. There are no data available to support the use of either drug when drug resistance has been documented. Due to the high pill burden of a M/XDR-TB regimen, the authors do not recommend the use of either drug in a M/XDR-TB regimen if drug resistance has been documented by a quality controlled laboratory.

MIC testing in Sweden

MICs for consecutive clinical isolates from the Karolinska University Hospital in Sweden as well as *M. tuberculosis* H37Rv ATCC 27294 were determined using the MGIT at Malmö University Hospital as previously described.[2] The following antibiotics and concentration ranges in µg/ml were tested

using two-fold dilutions, including the CC were applicable: isoniazid (0.016-0.25+0.1), rifampicin (0.016-1), ethambutol (1-4+5), pyrazinamide (8-128+100), amikacin (0.125-2), capreomycin (0.25-4), and levofloxacin (0.064-2). A small number of non-consecutive, resistant isolates were included as well, for which MIC testing was done with a two-fold concentration series above the CC.

SUPPLEMENTARY RESULTS

Cycloserine

Loss-of-function mutations in alanine dehydrogenase (Ald) have been shown to result in elevated MICs, which should apply to the XDR isolate 13739-13 as a result of a premature stop at codon 271 out of the total 371 codons.[3] Another XDR isolate (811-15) had an *ald* mutation at codon 198, which directly interacts with its coenzyme nicotinamide adenine dinucleotide (NAD).[4] Moreover, codon 238, which was mutated in a third isolate, is within 4 Å of the NAD binding region.[4]

PAS

For the isolates, for which PAS was tested, all 13 isolates that lacked mutations in any of the known PAS resistance genes tested susceptible at 4 μg/ml. Only five of the seven isolates with known resistance mutation in either the dihydrofolate synthase FolC, which is required for the activation of the PAS, or with promoter-up mutations in Rv2671, an alternative dihydrofolate reductase and one of the targets of PAS, tested resistant.[5-7] The susceptible result for 13255-14 (C-12T *Rv2671*) and 13739-13 (*folC* S150G) likely represented a false-susceptible result.[6-9] The remaining four isolates harboured two different amino acid changes at the same codon (P253A or P253L) of the thymidylate synthase ThyA, which is typically a signal for positive selection, yet all tested susceptible (repeatedly, in case of the former mutation).[10] This was surprising given that the latter mutation coincided with a promoter mutation in the second thymidylate synthase ThyX, which is believed to compensate for loss-of-function mutations in *thyA*, which, in turn, confer resistance by reducing the catalytic demand of the dihydrofolate reductase DfrA, the main target of PAS.[5, 11]

Table S1

Details of 25 patients in this study.

**Patient characteristic**	**N (%) or median (IQR)**
Age at admission	39 years (27.8-45.8 years)
MDR	20 (80)
XDR	5 (20)
Male gender	15 (62.5)
Non-German origin	22 (91.7)
Smear positive at diagnosis	13 (54.2)
Smear grade 3 or 4	6 (46.1)
Median pre-admission treatment time	19.5 days (10.8-82.5 days)
Time to culture positivity at baseline	17 days (8-28 days)
Time to culture positivity at week 1	22 days (12-40 days)

Table S2 (provided as separate file)

Table S3

Comparison of standard-algorithm derived regimens based on molecular DST with pDST.

Molecular DST assays	Mean overlap with pDST-derived regimen (95% CI)
WGS	93% (88-98%)
LPAs without ethambutol and pyrazinamide	87% (80-94%)
LPAs	63% (56-70%)
Xpert without ethambutol and pyrazinamide	68% (56-80%)
Xpert	49% (39-59%)

Table S4

Overview of the number of drugs administered in regimens based on molecular DST to which pDST showed resistance.

Molecular DST-derived regimen	Drugs used with pDST confirmed resistance [n]
WGS	0
LPAs without ethambutol and pyrazinamide	11
LPAs	56
Xpert without ethambutol and pyrazinamide	31
Xpert	77

Table S5

Number of antibiotics prescribed as part of the regimens based on either pDST, WGS, LPAs, or Xpert, stratified by the group of anti-TB drug, as defined in the WHO treatment guidelines.[12]

	Group A	Group B	A+B	Group C	Group D1	Group D2	Group D3	Total D	Total
pDST	18	19	37	49	8	7	9	24	110
WGS	16	19	35	47	4	10	10	24	106
LPAs	18	19	37	51	50	7	8	65	152
Xpert	25	25	50	50	50	0	0	50	150
p-values pDST vs. Xpert	<0.001*	<0.001*	<0.001*	0.84	<0.001*	<0.001*	<0.001*	<0.001*	<0.001*
p-values pDST vs. LPAs	1	1	1	0.69	<0.001*	1	0.668	<0.001*	<0.001*
p-values pDST vs. WGS	0.4047	1	0.54	0.69	0.0371*	0.1573	0.683	1	0.62

*$P < 0.05$ considered as significant using STATA (Differences of proportion).

Table S6

33 resistance genes analysed using WGS.[13]

H37Rv gene	Genomic start	Genomic stop	Rv number	associated antibiotic resistance
gyrB upstream	4998	5239		moxifloxacin, ofloxacin, levofloxacin
gyrB	5240	7267	Rv0005	moxifloxacin, ofloxacin, levofloxacin
gyrA upstream	7268	7301		moxifloxacin, ofloxacin, levofloxacin
gyrA	7302	9818	Rv0006	moxifloxacin, ofloxacin, levofloxacin
mshA upstream	575301	575347		isoniazid, ethionamide, prothionamide
mshA	575348	576790	Rv0486	isoniazid, ethionamide, prothionamide
rpoB upstream	759310	759806		rifampicin, rifabutin
rpoB	759807	763325	Rv0667	rifampicin, rifabutin
rpsL upstream	781312	781559		streptomycin
rpsL	781560	781934	Rv0682	streptomycin
rplC upstream	800793	800808		linezolid
rplC	800809	801462	Rv0701	linezolid
tap	1406081	1407340	Rv1258c	streptomycin, PAS
tap upstream	1407341	1408238	Rv1259	streptomycin, PAS
rrs upstream	1471743	1471845		streptomycin, amikacin, kanamycin, capreomycin
rrs	1471846	1473382	Rvnr01	streptomycin, amikacin, kanamycin, capreomycin
rrl upstream	1473383	1473657		linezolid, capreomycin, clarithromycin
rrl	1473658	1476795	Rvnr02	linezolid, capreomycin, clarithromycin
fabG1 upstream	1673300	1673439		isoniazid, ethionamide, prothionamide
fabG1	1673440	1674183	Rv1483	isoniazid, ethionamide, prothionamide
inhA upstream	1674184	1674201		isoniazid, ethionamide, prothionamide
inhA	1674202	1675011	Rv1484	isoniazid, ethionamide, prothionamide
tlyA	1917940	1918746	Rv1694	capreomycin
cycA	1929786	1931456	Rv1704c	cycloserine
cycA upstream	1931457	1932654		cycloserine
katG	2153889	2156111	Rv1908c	isoniazid
katG upstream	2156112	2156148		isoniazid
furA	2156149	2156592	Rv1909c	isoniazid
furA upstream	2156593	2156705		isoniazid
pncA	2288681	2289241	Rv2043c	pyrazinamide
pncA upstream	2289242	2289281		pyrazinamide

eis	2714124	2715332	Rv2416c	kanamycin
eis upstream	2715333	2715471		kanamycin
folC	2746135	2747598	Rv2447c	PAS
Rv2671 upstream	2985731	2986838	Rv2670c	PAS
Rv2671	2986839	2987615	Rv2671	PAS
dfrA	3073130	3073609	Rv2763c	PAS, co-trimoxazole
dfrA upstream	3073610	3073679		PAS, co-trimoxazole
thyA	3073680	3074471	Rv2764c	PAS
thyA upstream	3074472	3074635		PAS
ald upstream	3086755	3086819		cycloserine
ald	3086820	3087935	Rv2780	cycloserine
whiB7	3568401	3568679	Rv3197A	streptomycin, kanamycin, clarithromycin, PAS
whiB7 upstream	3568680	3569108		streptomycin, kanamycin, clarithromycin, PAS
alr	3840194	3841420	Rv3423c	cycloserine
alr upstream	3841421	3841713		cycloserine
aftA,embC	4237932	4243147	Rv3792,Rv3793	ethambutol
embA upstream	4243148	4243232		ethambutol
embA,embB	4243233	4249810	Rv3794,Rv3795	ethambutol
ubiA	4268925	4269833	Rv3806c	ethambutol
ubiA upstream	4269834	4269839		ethambutol
ethA	4326004	4327473	Rv3854c	ethionamide, prothionamide, thioacetazone
ethA upstream	4327474	4327548		ethionamide, prothionamide, thioacetazone
gidB	4407528	4408202	Rv3919c	streptomycin
gidB upstream	4408203	4408333		streptomycin

Table S7

Mutations in resistance genes that were considered not to confer resistance.

**Genome Pos (H37Rv)**	**polymorphism**	**Gene**	**phylogenic marker for**
7362	E21Q (gag/Cag)	Rv0006	non H37Rv_lab_strain
7585	S95T (agc/aCc)	Rv0006	non H37Rv_like
8040	G247S (ggc/Agc)	Rv0006	LAM subgroup
9304	G668D (ggc/gAc)	Rv0006	non H37Rv_like
157292	E103E (gag/gaa)	Rv0129c	LAM
403364	P826P (ccc/cct)	Rv0338c	LAM subgroup
491742	F320F (ttt/ttC)	Rv0407	non Euro-American (lineage 4)
497491	D270D (gac/gat)	Rv0411c	Beijing
575907	A187V (gca/gTa)	Rv0486	Beijing subgroup
648856	G107G (ggt/ggc)	Rv0557	non Euro-American (lineage 4)
763031	A1075A (gct/gcC)	Rv0667	non Euro-American (lineage 4)

764995	A542A (gcc/gcG)	Rv0668	LAM
766645	E1092D (gaa/gaC)	Rv0668	Beijing subgroup
775639	I948V (att/Gtt)	Rv0676c	non H37Rv_lab_strain
776100	T794I (acc/aTc)	Rv0676c	non Euro-American (lineage 4)
776182	D767N (gac/Aac)	Rv0676c	Beijing subgroup
781395	781395 t/c	rpsL upstream	non H37Rv_lab_strain
797736	L268L (ctc/ctt)	Rv0697	Beijing subgroup
931123	Y57Y (tat/tac)	Rv0835	non Euro-American (lineage 4)
1474001	344 c/t	Rvnr02	H37Rv_like subgroup
1762615	A291V (gcc/gTc) (0)	Rv1557	LAM subgroup
1834177	R212R (cga/cgC)	Rv1630	Beijing
1917972	L11L (cta/ctG)	Rv1694	non H37Rv_lab_strain
1931179	R93L (cgg/cTg)	Rv1704c	non H37Rv_lab_strain
2053682	I80I (atc/att)	Rv1811	LAM
2154724	R463L (cgg/cTg)	Rv1908c	non Euro-American (lineage 4)
2505085	A205A (gcc/gct)	Rv2231c	Beijing subgroup
2518919	G269S (ggt/Agt)	Rv2245	LAM subgroup
2714846	V163I (gtc/Atc)	Rv2416c	Beijing subgroup
2752122	T20R (acg/agg)	Rv2450c	Beijing
2955957	Asp64Ala (gat/gct)	Rv2629	Beijing
3073868	T202A (acc/Gcc)	Rv2764c	LAM
3326554	N152H (aac/cac)	Rv2971	non Euro-American (lineage 4)
3336825	T365A (aca/Gca)	Rv2981c	non H37Rv_like
3566107	A107D (gcc/gAc)	Rv3196	Beijing subgroup
3836739	D51D (gac/gat)	Rv3417c	H37Rv_like subgroup
4238675	G248G (ggc/ggT)	Rv3792	Beijing subgroup
4242643	R927R (cgc/cgT)	Rv3793	non H37Rv_lab_strain
4243346	Q38Q (caa/caG)	Rv3794	Beijing subgroup
4243460	C76C (tgc/tgT)	Rv3794	Beijing
4248115	D534D (gac/gaT)	Rv3795	Beijing subgroup
4407588	A205A (gca/gcG)	Rv3919c	non Euro-American (lineage 4)
4407927	E92D (gaa/gaC)	Rv3919c	Beijing
4408156	L16R (ctt/cGt)	Rv3919c	LAM

Table S8 (provided as separate file)

References

1. Horsburgh CR, Jr., Barry CE, 3rd, Lange C. Treatment of tuberculosis. *N Engl J Med* 2015; **373**: 2149-60.
2. Sturegård E, Ängeby KA, Werngren J et al. Little difference between minimum inhibitory concentrations of *Mycobacterium tuberculosis* wild-type organisms determined with BACTEC MGIT 960 and Middlebrook 7H10. *Clin Microbiol Infect* 2015; **21**: 148 e5-7.
3. Desjardins CA, Cohen KA, Munsamy V et al. Genomic and functional analyses of *Mycobacterium tuberculosis* strains implicate *ald* in D-cycloserine resistance. *Nat Genet* 2016; **48**: 544-51.
4. Tripathi SM, Ramachandran R. Crystal structures of the *Mycobacterium tuberculosis* secretory antigen alanine dehydrogenase (Rv2780) in apo and ternary complex forms captures "open" and "closed" enzyme conformations. *Proteins* 2008; **72**: 1089-95.
5. Moradigaravand D, Grandjean L, Martinez E et al. *dfrA thyA* double deletion in *para*-aminosalicylic acid-resistant *Mycobacterium tuberculosis* Beijing strains. *Antimicrob Agents Chemother* 2016; **60**: 3864-7.
6. Zheng J, Rubin EJ, Bifani P et al. *Para*-aminosalicylic acid is a prodrug targeting dihydrofolate reductase in *Mycobacterium tuberculosis*. *J Biol Chem* 2013; **288**: 23447-56.
7. Zhao F, Wang XD, Erber LN et al. Binding pocket alterations in dihydrofolate synthase confer resistance to *para*-aminosalicylic acid in clinical isolates of *Mycobacterium tuberculosis*. *Antimicrob Agents Chemother* 2014; **58**: 1479-87.
8. Zhang X, Liu L, Zhang Y et al. Genetic determinants involved in *p*-aminosalicylic acid resistance in clinical isolates from tuberculosis patients in northern China from 2006 to 2012. *Antimicrob Agents Chemother* 2015; **59**: 1320-4.
9. Cheng VW, Leung KS, Kwok JS et al. Phylogenetic and structural significance of dihydrofolate synthase (*folC*) mutations in drug-resistant *Mycobacterium tuberculosis*. *Microb Drug Resist* 2016.
10. Farhat MR, Shapiro BJ, Kieser KJ et al. Genomic analysis identifies targets of convergent positive selection in drug-resistant *Mycobacterium tuberculosis*. *Nat Genet* 2013; **45**: 1183-9.
11. Merker M, Kohl TA, Roetzer A et al. Whole genome sequencing reveals complex evolution patterns of multidrug-resistant *Mycobacterium tuberculosis* Beijing strains in patients. *PLoS One* 2013; **8**: e82551.
12. World Health Organization. WHO treatment guidelines for drug-resistant tuberculosis. 2016 update. http://www.who.int/tb/MDRTBguidelines2016.pdf (accessed 3.8.2016).
13. Köser CU, Bryant JM, Becq J et al. Whole-genome sequencing for rapid susceptibility testing of *M. tuberculosis*. *N Engl J Med* 2013; **369**: 290-2.
